# Genomic Signatures of Immune Activation Predict Outcome in Advanced Stages of Ovarian Cancer and Basal-Like Breast Tumors

**DOI:** 10.3389/fonc.2019.01486

**Published:** 2020-01-10

**Authors:** Ana Alcaraz-Sanabria, Mariona Baliu-Piqué, Cristina Saiz-Ladera, Katerin Rojas, Aránzazu Manzano, Gloria Marquina, Antonio Casado, Francisco J. Cimas, Pedro Pérez-Segura, Atanasio Pandiella, Balázs Gyorffy, Alberto Ocana

**Affiliations:** ^1^Translational Oncology Laboratory, Centro Regional de Investigaciones Biomedicas, Castilla-La Mancha University, Albacete, Spain; ^2^Experimental Therapeutics Unit, Medical Oncology Department, Hospital Clínico San Carlos, Instituto de Investigación Sanitaria (IdISSC) and CIBERONC, Madrid, Spain; ^3^Instituto de Biología Molecular y Celular del Cáncer and CIBERONC, CSIC, Salamanca, Spain; ^4^Departments of Bioinformatics and Pediatrics, Semmelweis University, Budapest, Hungary; ^5^MTA TTK Lendület Cancer Biomarker Research Group, Institute of Enzymology, Budapest, Hungary

**Keywords:** ovarian cancer, breast cancer, genomic signatures, immune infiltrates, biomarkers

## Abstract

There is an unmet need for new therapies in metastatic ovarian cancer and basal-like breast cancer since no curative therapies are currently available. Immunotherapy has shown to be active in several solid tumors, but particularly more in those where a pre-activated immune state does exist. In this work, we aim to identify biomarkers that could distinguish immune-activated tumors and predict response to therapies in ovarian and basal-like breast cancer, as well as their association with the level of tumor immune infiltration. We found that the combined expression of IFNG, CD30, CXCL13, and PRF1 correlated with better overall survival (OS) in advanced stage ovarian cancer. This was confirmed using an independent dataset from TCGA. Interestingly, we observed that this gene combination also predicted for better prognosis in ovarian tumors with low mutational load, which typically respond less to immunotherapy. Expression of IFNG, CD30, CXCL13, and PRF1 was associated with increased level of immune infiltrates (CD8^+^ T cells, dendritic cells, and neutrophils) within the tumor. Moreover, we found that these gene signature also correlated with an increased OS and with a higher level of tumor immune infiltrates (B cells, CD8^+^ T cells, CD4^+^ T cells, neutrophils, and dendritic cells) in basal-like breast cancer. In conclusion, our analysis identifies genes signatures with potential to recognize immune activated ovarian and basal-like breast cancers with favorable prognosis and with a remarkable predictive capacity in tumors with low mutational burden. The presented results led to a hypothesis being formulated, but prospective clinical studies are needed to support a potential clinical application.

## Introduction

Ovarian cancer is still an incurable disease in its late stage (1, 2). Several therapies have been explored and some have reached the clinical setting, improving survival, but not eradicating the disease (1, 2). Among different strategies can be included agents that act on DNA repair like the PARP inhibitors Olaparib, Niraparib, and Rucaparib, antiangiogenic antibodies like bevacizumab or novel chemotherapeutics that affect cell division such as trabectedin (1–3). Even with the incorporation of these therapies metastatic ovarian cancer is still an incurable disease and therefore novel therapeutics are necessary (2). Furthermore, how to integrate these new compounds in the clinic is challenging and indeed, most of them have been developed in combination with approved chemotherapies intending to augment their efficacy (2).

Basal-like breast tumors, which represent around 15% of all breast cancer cases, are characterized by its poor prognosis and limited therapeutic opportunities (4). As described by gene expression analyses, the majority of tumors composing the basal-like breast cancer subtype are triple negative breast cancers (TNBC). This subtype is represented by tumors that lack the presence of hormone receptors and HER2 overexpression on the cell membrane (4). Basal-like breast tumors share some biological characteristics with ovarian cancers, such as a high genomic instability, and their inability to repair DNA, reflected in the presence of mutations in BRCA1/2 genes (4, 5).

Immunotherapy has gained momentum, as agents that modulate the T cell response by augmenting their activity have shown clinical efficacy in a wide range of solid tumors (6). Check-point inhibitors, such as PD1/PDL1 and CTLA-4 inhibitors, prevent T-cell inhibition and promote the activation and function of T cells (6). Antibodies against these receptors have shown promising therapeutic outcomes in lung, kidney, or bladder cancer among other tumor types and have been approved for certain cancer treatments (7). However, it has been shown that for these agents to obtain a clinical response, tumors must be in a pre-activated immune state, the so called hot tumors (7–10). Late stage ovarian cancer and basal-like breast cancer are characterized by high genetic instability, neo-antigen expression and are typically prone to respond to chemotherapy (9, 11, 12). Check-point inhibitors have not yet reached the clinical setting in ovarian cancer, but in TNBC check-point inhibitors improve survival when combined with chemotherapy (13).

A main task in ovarian and basal-like breast cancers is the discovery of biomarkers that identify immune activated tumors and that predict response to therapy (14). With this aim, here we used established transcriptomic immune signatures to identify ovarian and basal-like breast cancer patients with favorable prognosis. We found that, in ovarian cancer, the expression of CXCL13, INFG, CD30, and PRF1 genes predicted favorable prognosis, and that CXCL3, INFG, and PRF1 were associated with an increased infiltration level of CD8^+^ T cells and dendritic cells and neutrophils within the tumor. We observed similar results in basal-like breast tumors suggesting the potential application of these gene signatures in ovarian and basal-like breast cancers to identify a subpopulation of patients with better prognosis.

## Materials and Methods

### Validated Transcriptomic Immune Signatures

We used three previously described gene sets, the IFN gamma signature (1), the expanded immune gene signature (1), the cytotoxic T lymphocyte (CTL) signature (2, 15), and the HLA-A and HLA-B genes as they have been associated with prognosis (16) to study the relation of the genes composing the different signatures with patient clinical outcomes in ovarian and basal-like breast cancer.

### Outcome Analysis in Kaplan Meier (KM) Plotter

The KM Plotter Online Tool (http://www.kmplot.com) (3, 4), a publicly available database, was used to study the relationship between the expression of different genes from the validated immune signatures and patient clinical outcomes for ovarian cancer. We evaluated the prognostic value of mRNA expression of each gene composing the different immune signatures and several gene combinations according to progression free survival (PFS) and overall survival (OS) in ovarian cancer patients with early (I and II) and advanced stage (III and IV) and in basal-like breast cancer tumors (all stages). For Ovarian cancer stage III and IV, the following datasets were used for the analysis: GSE14764, GSE15622, GSE18520, GSE19829, GSE23554, GSE26193, GSE26712, GSE27651, GSE30161, GSE3149, GSE51373, GSE63885, GSE65986, GSE9891, and TGCA. A total of 1,220 patients were included in those datasets, from which 1,099 were treated with Platin, 720 with Taxol, 703 with the combination of Platin+Taxol, 47 with Bevacizumab, 101 with Docetaxel, 19 with Gemcitabine, 212 with Paclitaxel, and 114 with Topotecan. The KM Plotter Online Tool was also used to compare the predictive value of several gene combinations in patients with low and high mutational burden.

Patients were divided into two groups, high vs. low expression, based on the best cut-off values of gene expression (smallest *p*-value). The best cut-off values were determined by algorithms embedded in KM plotter. The OS KM plots are presented with the hazard ratio (HR), the 95% confidence interval (CI) and the log-rank *p*-value.

### Tumor Infiltrating Immune Cells Association With Gene Expression in Tumor Immune Estimation Resource (TIMER) Platform

The association between the abundance of tumor immune infiltrates (B-cells, CD4^+^ T-cells, CD8^+^ T-cells, dendritic cells, macrophages, and neutrophils) and the expression of the selected genes was analyzed via the Tumor Immune Estimation Resource (TIMER) platform (5, 6), a web server which contains 10,897 samples from diverse cancer types available in the TCGA database. The correlation graphics show the purity-corrected partial Spearman's correlation and its statistical significance.

## Results

### Selection of Immunologic Signatures

We first selected immunologic signatures previously described to be associated with clinical outcome in several indications, such as melanoma, mainly after receiving treatment with check-point inhibitors (15, 17–19). We selected the IFN gamma signature, the expanded immune gene signature, the cytotoxic T lymphocyte (CTL) signature and the expression of MHC class I molecules HLA-A and HLA-B, as all of them have been described to be associated with prognosis in solid tumors (15, 17–19) (Table 1).

**Table 1:** Immune signatures used in this study including HLA A and B, IFN gamma signature, expanded immune gene signature, and cytotoxic T lymphocyte (CTL) signature, and their corresponding genes.

**Signature**	**Genes**
HLA	HLA-A, HLA-B
IFN gamma signature	IDO1, CXCL10, CXCL9, HLA-DRA, ISGF-3, IFNG
Expanded immune gene signature	CD30, IDO1, CIITA, CD3E, CCL5, GZMK, CD2, HLA-DRA, CXCL13, IL3RG, NKG7, HLA-E, CXCR6, LAG3, TAGAP, CXCL10, STAT1, GZMB
Cytotoxic T lymphocyte (CTL) level signature	CD8A, CD8B, GZMA, GZMB, PRF1

### Association of Described Signatures With Progression Free Survival and Overall Survival in Early Stage Ovarian Cancer

**Figure 1:**
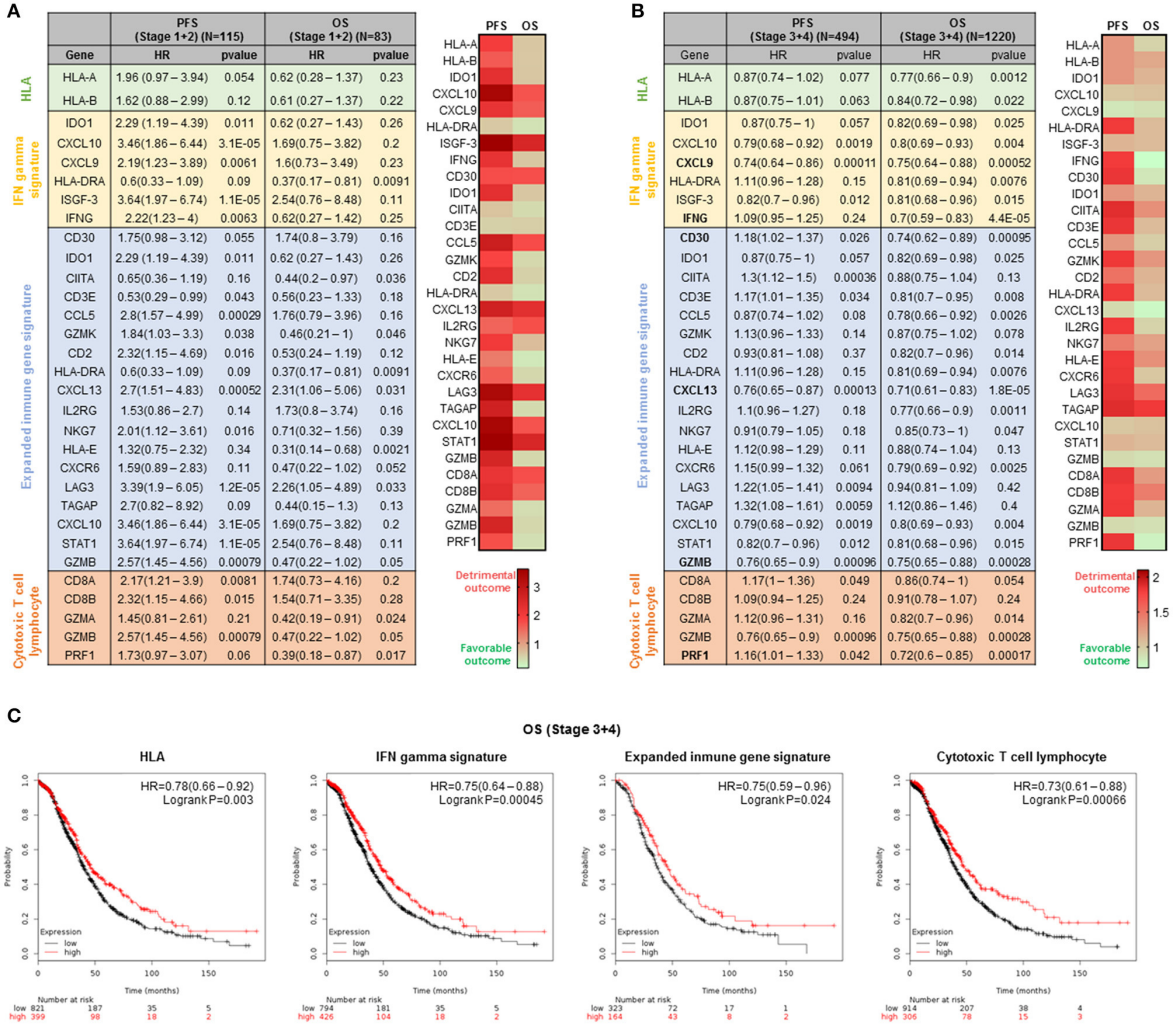
Prognosis analysis for ovarian cancer stage I+II and III+IV based on the expression of genes from the four immune signatures. **(A)** Table of the association of the expression of the genes composing the four immune signatures with progression free survival (PFS) and overall survival (OS) in stage I and II ovarian cancer and the heatmap that summarized the information contained in the table. **(B)** Association of the expression of the genes composing the four immune signatures with progression free survival (PFS) and overall survival (OS) in stage III+IV ovarian cancer and heatmap summarizing the information from the table. The tables show the hazard ratio (HR) and the Kaplan-Meier *p*-value for the individual genes of the four immune signatures. The signatures which predicted favorable outcome are highlighted in green and those which predicted detrimental outcome are highlighted in red. **(C)** Kaplan-Meier survival plots showing the association between the four immune signatures and OS. The hazard ratio (HR) and the Kaplan-Meier *p*-value are shown.

We used the Kaplan Meier (KM) plotter online tool (20) to associate the presence of genomic transcripts with progression free survival (PFS) and overall survival (OS) in ovarian cancer patients. First, we selected early stage patients, including stage I and II. For most of these patients (stage IA and IB) surgical removal of the tumor is the only therapeutic approach and no additional medical therapy is necessary (2). In these subgroup of ovarian cancer patients, we found that a large number of genes from the selected immunologic signatures were associated with detrimental PFS. Contrary to PFS, we observed that for OS a large number of transcripts were linked with a favorable outcome (Figure 1A).

### Identification of Transcripts Linked With Overall Survival in Advanced Stage Ovarian Cancer

Then, we then focused on the advanced stage ovarian cancer, as it is the condition with higher prevalence and worse prognosis (2). When exploring stage III and IV, the findings for PFS were ambiguous with some genes predicting for favorable and others for detrimental outcome (Figure 1B). Per contra, for OS, we found that the expression of most genes was associated with improved survival (Figure 1B). Twenty-four transcripts predicted for good prognosis and only seven for detrimental outcome. This observation suggests that the presence of an immune activated state is associated with better long-term survival in advanced stage ovarian cancer. As shown in Figure 1C, the combined analysis of each signature did not improve prediction compared with some of the transcripts alone, i.e., INFG and CXCL13: IFN gamma signature (HR = 0.75; CI = 0.64–0.88; log rank *p* = 0.00045), the expanded immune gene signature (HR = 0.75; CI = 0.59–0.96; log rank *p* = 0.024), the cytotoxic T lymphocyte (CTL) signature (HR = 0.73; CI = 0.61–0.88; log rank *p* = 0.00066), or the expression of MHC class I molecules HLA-A and HLA-B (HR = 0.78; CI = 0.66–0.92; log rank *p* = 0.003).

### Combined Signatures Predict Outcome in Advanced Stage Ovarian Cancer

**Figure 2:**
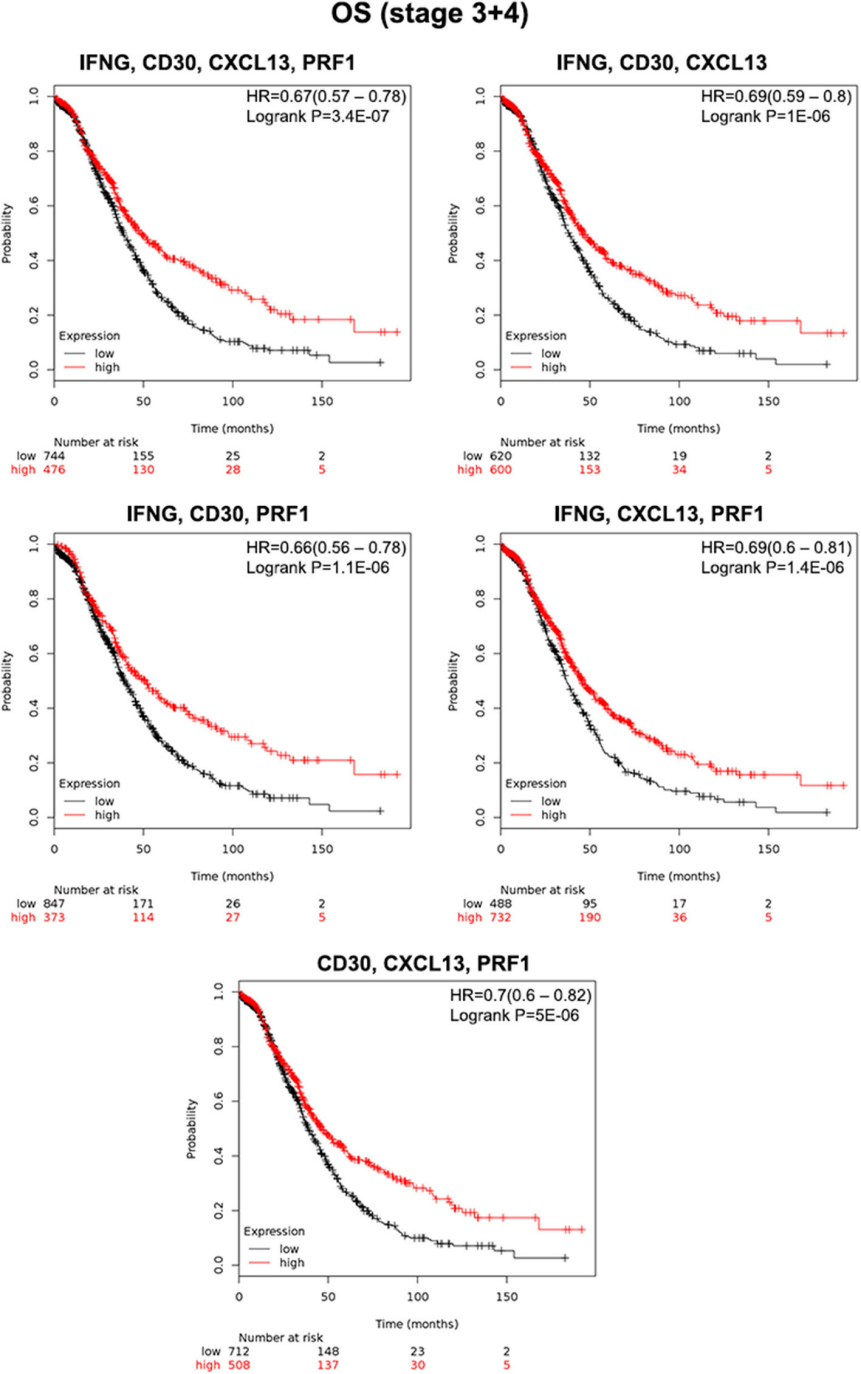
Association of the gene combinations with overall survival (OS) in ovarian cancer stage III and IV. Kaplan-Meier survival plots showing the association between the combined gene expression levels (IFNG, CD30, CXCL13, PRF1; IFNG, CD30, CXCL13; IFNG, CD30, PRF1; IFNG, CXCL13, PRF1; and CD30, CXCL13, PRF1) and prognosis (OS) in patients from stages III and IV. The hazard ratio (HR) and the Kaplan-Meier *p*-value are shown.

Next we explored whether gene combinations would be able to predict survival with a higher strength compared to single transcripts. Multiple testing enable us the selection of four gene combinations that predicted favorable outcome better than single transcripts: IFNG, CD30, CXCL13, and PRF1 (HR = 0.67; CI = 0.57–0.78; log rank *p* = 3.4E-07); IFNG, CD30 and CXCL13 (HR = 0.69; CI = 0.59–0.8; log rank *p* = 1.1E-06); IFNG, CD30, and PRF1 (HR = 0.66; CI = 0.56–0.78; log rank *p* = 1 E-06); IFNG, CXCL13 and PRF1 (HR = 0.69; CI = 0.6–0.81; log rank *p* = 1.4E-06), and CD30, CXCL13, and PRF1 (HR = 0.7; CI = 0.6–0.82; log rank *p* = 5E-06) (Figure 2).

### Association of IFNG, CXCL13, CD30, and PRF1 With Survival in Ovarian Tumors With Low Mutational Load

**Figure 3:**
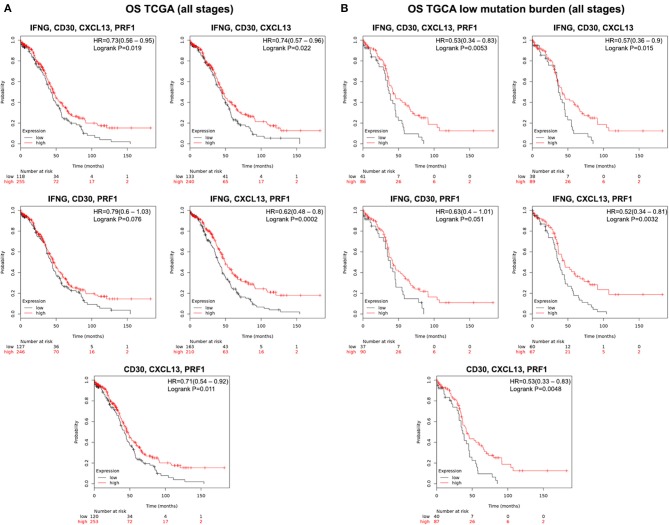
Validation cohort using data from TCGA and predictive value of the five gene combination when selecting for low mutation burden in ovarian cancer patients from all stages. **(A)** Kaplan-Meier survival plots showing the association between the combined gene expression levels (IFNG, CD30, CXCL13, PRF1; IFNG, CD30, CXCL13; IFNG, CD30, PRF1; IFNG, CXCL13, PRF1; and CD30, CXCL13, PRF1) and overall survival (OS) in ovarian cancer patients from all stages. **(B)** Survival plots depicting the association between the combined gene expression levels with low mutational load and patient outcome (OS). The hazard ratio (HR) and the Kaplan-Meier *p*-value are shown.

To confirm our results, first we performed an independent validation using a TCGA dataset. We confirmed that, even in a smaller cohort of patients, our four gene combinations were able to predict for increased OS (Figure 3A). Thereafter, we explored whether these gene signatures were also able to predict outcome in a cohort of tumors with low and high mutational load. We focused on this subgroup as tumors with low mutational load have shown to respond less to current immunotherapies. We found that in the cohort of patients harboring tumors with low mutational load the four signatures predicted better outcome (Figure 3B): IFNG, CD30, CXCL13, and PRF1 (HR = 0.53; CI = 0.34–0.83; log rank *p* = 0.0053); IFNG, CD30, and CXCL13 (HR = 0.57; CI = 0.36–0.90; log rank *p* = 0.015); IFNG, CD30, and PRF1 (HR = 0.63; CI = 0.4–1.01; log rank *p* = 0.051); IFNG, CXCL13, and PRF1 (HR = 0.52; CI = 0.34–0.81; log rank *p* = 0.0032); CD30, CXCL13, and PRF1 (HR = 0.53; CI = 0.33–0.83; log rank *p* = 0.0048). On the other hand, we observed that these gene signatures did not predict for improved OS and were even associated with poor prognosis in a cohort of ovarian tumors with high mutational burden (Supplementary Figure 1).

### Correlation of IFNG, CXCL13, CD30, and PRF1 With Tumor Immune Infiltrates

**Figure 4:**
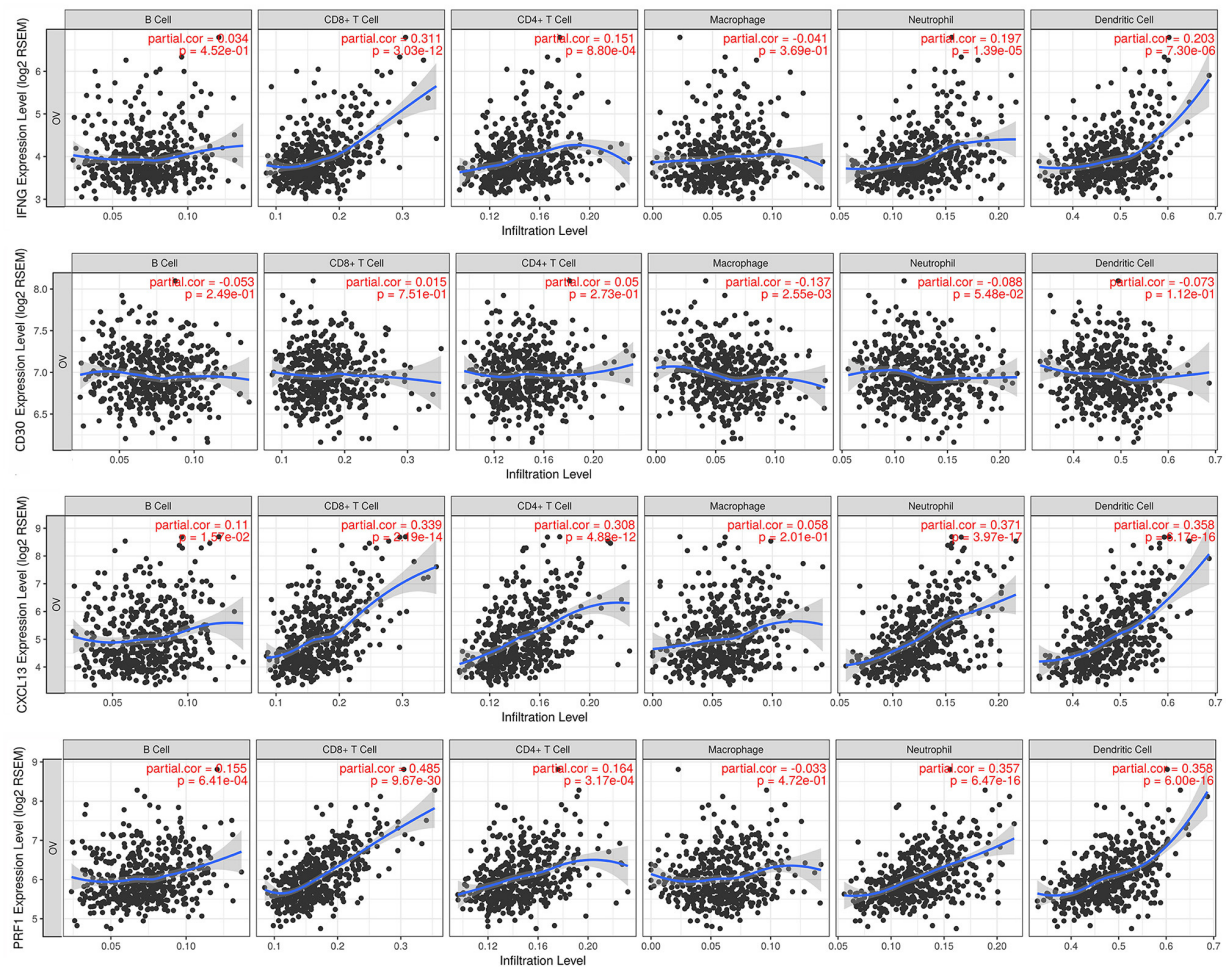
Association of the expression of the four selected genes with immune infiltrates in ovarian cancer. Partial correlation analysis between genes expression (IFNG, CD30, CXCL13, and PRF1) and the level of tumor immune infiltrates (B-cells, CD8^+^ T cells, CD4^+^ T cells, macrophages, neutrophils and dendritic cells).

We finally explored the association of the expression at a transcriptomic level of IFNG, CD30, CXCL13, and PRF1 with the presence of tumoral infiltrating immune cell populations. We found that the expression level of IFNG correlated with the presence of CD8^+^ T cells (part.cor = 0.311) and dendritic cells (part.cor = 0.203); the expression level of CXCL13 correlated with the infiltration level of CD8^+^ T cells (part.cor = 0.339), CD4^+^ T cells (part.cor = 0.308), dendritic cells (part.cor = 0.358), and neutrophils (part.cor = 0.371); and finally, that the expression level of PRF1 was correlated with the infiltration level of CD8^+^ T cells (part.cor = 0.485), dendritic cells (part.cor = 0.358) and neutrophils (part.cor = 0.357) (Figure 4). For CD30 we found no association between its expression level and the level of immune infiltrates for any of the populations explored, including B cells (Figure 4). None of the studied genes was associated with changes in the presence of macrophages in the tumor.

### Combined Signatures, Survival and Immune Infiltrates in Basal-Like Breast Cancer

**Figure 5:**
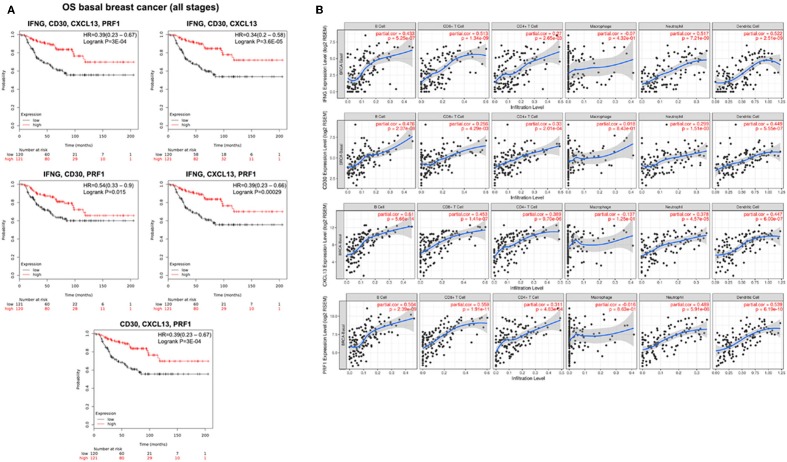
Prognosis analysis and immune infiltration level for basal like breast tumors. **(A)** Kaplan-Meier survival plots showing the association between the combined gene expression levels (IFNG, CD30, CXCL13, PRF1; IFNG, CD30, CXCL13; IFNG, CD30, PRF1; IFNG, CXCL13, PRF1; and CD30, CXCL13, PRF1) and overall survival (OS) in basal breast cancer patients. The hazard ratio (HR) and the Kaplan-Meier *p*-value are shown. **(B)** Association of the expression of the four selected genes with immune infiltration levels. Partial correlation analysis between genes expression (IFNG, CD30, CXCL13, PRF1) and the level of tumor immune infiltrates (B-cells, CD8^+^ T cells, CD4^+^ T cells, macrophages, neutrophils, and dendritic cells).

Given the fact that basal-like breast cancer tumors share many biological characteristics with ovarian cancer (4), we decided to explore the capacity of the identified immune signatures to predict survival in basal-like breast tumors. We observed a clear association of our gene signatures with favorable prognosis that was higher than for ovarian cancer: IFNG, CD30, CXCL13, and PRF1 (HR = 0.39; CI = 0.23–0.67; log rank *p* = 3E-04); IFNG, CD30, and CXCL13 (HR = 0.34; CI = 0.2–0.58; log rank *p* = 3.6E-05); IFNG, CD30, and PRF1 (HR = 0.54; CI = 0.33–0.9; log rank *p* = 0.015); IFNG, CXCL13, and PRF1 (HR = 0.39; CI = 0.23–0.66; log rank *p* = 0.00029); CD30, CXCL13 and PRF1 (HR = 0.39; CI = 0.23–0.67; log rank *p* = 3E-04) (Figure 5A).

Then, we studied whether in basal-like breast cancer the expression of these genes was also associated with increased infiltration of immune cells. We found that the expression level of the four studied genes was associated with higher infiltrates of most immune cells (B cells, CD8^+^ T cells, CD4^+^ T cells, neutrophils and dendritic cells), with the exception of macrophages. IFNG expression levels correlated with the presence of B-cells (part.cor = 0.433), CD8^+^ T cells (part.cor = 0.513), CD4^+^ T cells (part.cor = 0.27), neutrophils (part.cor = 0.517), and dendritic cells (part.cor = 0.522); the expression level of CXCL13 correlated with the infiltration level of B-cells (part.cor = 0.61), CD8^+^ T cells (part.cor = 0.453), CD4^+^ T cells (part.cor = 0.389), neutrophils (part.cor = 0.378), and dendritic cells (part.cor = 0.447); finally, the expression level of PRF1 was correlated with the infiltration level of B-cells (part.cor = 0.504), CD8^+^ T cells (part.cor = 0.559), CD4^+^ T cells (part.cor = 0.311), neutrophils (part.cor = 0.489), and dendritic cells (part.cor = 0.539). Unlike in ovarian cancer, we found that the expression level of CD30 correlated with the infiltration level of B-cells (part.cor = 0.476), CD8^+^ T cells (part.cor = 0.25), CD4^+^ T cells (part.cor = 0.33), neutrophils (part.cor = 0.299), and dendritic cells (part.cor = 0.449) (Figure 5B).

## Discussion

Tumors with a pre-activated immune state typically have a more favorable outcome and are likely to respond better to chemotherapies or immunotherapies (6, 8). In the present article we used previously published gene signatures (12, 13) to describe immune signatures related to immune activation that identify patients with favorable OS prognosis in advanced stage ovarian cancer and basal-like breast tumors.

We found that the single transcripts from the IFN gamma, the expanded immune gene and the CTL signature, and the HLA-A and HLA-B molecules predicted for better OS. The predictive value of individual transcripts is confirmed when each whole signature is pooled together validating its potential utility. In a further step, we studied combinations of genes that would better predict outcome than the one achieved by single transcripts. The combination of the genes IFNG, CXCL13, CD30, and PRF1 clearly predicted favorable outcome in III and IV stage ovarian cancer and in basal-like breast cancer. The genes included in this signature clearly have an effector and chemotactic function. *IFNG* is a soluble cytokine secreted by innate and adaptive cells that binds to *INFG* receptor, activating the cellular response (21). PRF1 form membrane pores releasing granzymes leading to the cytolysis of the target cells (22, 23). CD30 is a cell membrane protein of the tumor necrosis factor receptor family and is expressed in effector T cells (24). CXCL13 is a chemotactic chemokine which plays a pivotal role in the recruitment and maintenance of an active population of B cells, T cells and another immune cells within the inflammatory side (25). Furthermore, it is a central chemokine for germinal center formation, which could be linked to the formation of tertiary lymphoid organs within the tumor.

The four genes composing the signatures have a relevant role in the activation and maintenance of effector T cell responses and may also be involved in attracting immune cells into the tumor tissue. To test this hypothesis, we used the TIMER online tool and found that the expression level of IFNG, CXCL13, CD30, and PRF1, positively correlated with the level of tumor infiltrating immune cells, including dendritic cells, and CD8^+^ T cells which are involved in the activation of the anti-tumor effector response. Presence of dendritic cells in the tumor is necessary for antigen presentation and activation of effector T cells that, both critical for the initiation and maintenance of an effective immune response against cancer cells (26, 27). This finding suggests that the combined gene signature could help identify tumors with higher number of immune infiltrates, i.e., “hot” tumors.

We find particularly relevant that the selected immune signatures predicted for better OS but not for better PFS and mainly in advanced disease, in patients with stage III and IV ovarian cancer. We hypothesize that these findings could be due to two main reasons; first the number of patients in early stage was significantly smaller than in the advanced stage disease (Stage I and II: *n* = 115 for PFS and 83 for OS; compared with *n* = 494 for PFS and 1,220 for OS stage III and IV); second, the fact that advanced stage tumors are more heterogeneous. In addition, advanced and metastatic tumors have more genetic instability, with a higher neoantigen production (9, 11, 12). In other indications, it has been shown that immunotherapies show clinical activity in tumors with genomic instability, identified by the presence of high microsatellite instability or mismatch repair deficiency (12, 28, 29); and that tumors with higher level of tumor infiltrating lymphocytes respond better to chemotherapy and are associated with favorable outcome (30, 31). The fact that advanced and metastatic tumors have more genetic instability, higher neoantigen production (9, 11, 12), and that metastatic cancer patients typically receive chemotherapy, which known to produce genomic instability (6); may explain the association between the immune gene signatures and OS in stages III and IV cancers but not in stage I and II cancers.

In basal-like breast tumors, a disease with very limited therapeutic options and in which immunotherapy has shown limited efficacy (13), our signatures were able to predict outcome in a higher manner than in ovarian cancer. In addition, as seen in ovarian cancer, there was also a clear link between the expression of IFNG, CXCL13, CD30, and PRF1 and presence of immune cells within the tumor, including B cells, CD8^+^ T cells, CD4^+^ T cells, dendritic cells, and neutrophils.

Our study has several limitations. First, gene selection in this study was based on previously described transcriptomic signatures, therefore we may have missed genes with a predictive role because they may not have been included in the studied signatures. Second, we are aware that this is an *in silico* analysis that needs further validation. Our study calls for an independent prospective clinical study to confirm the predictive capacity of our findings.

In conclusion, we described an immunologic transcriptomic signature associated with favorable outcome in late stage ovarian and basal-like cancer patients. Confirmation in prospective clinical studies is warranted.

## Data Availability Statement

Publicly available datasets were analyzed in this study. This data can be found here: https://kmplot.com/analysis/, http://cistrome.org/TIMER/download.html.

## Author Contributions

AO conceived the study and did the original design of the study. AA-S, MB-P, CS-L, KR, and AM searched the data and performed the analysis. AP, MB-P, CS-L, and AO wrote the manuscript. All authors reviewed, included modification, and approved the final version of the manuscript.

### Conflict of Interest

AO receives research funding from Entrechem, Servier and Daiichi-Sankyo and travel expenses from Merck. AP receives research funding from Daiichi-Sankyo. PP-S receives research funding from Merck and MSD. The remaining authors declare that the research was conducted in the absence of any commercial or financial relationships that could be construed as a potential conflict of interest.
