# Supplementary material for: Genomic Signatures of Immune Activation Predict Outcome in Advanced Stages of Ovarian Cancer and Basal-Like Breast Tumors
Source: Front Oncol. 2020 Jan 10;9:1486. doi: 10.3389/fonc.2019.01486 (PMC6965148; doi:10.3389/fonc.2019.01486)
Supplement: Supplementary file 1 [file Data_Sheet_1.PDF]

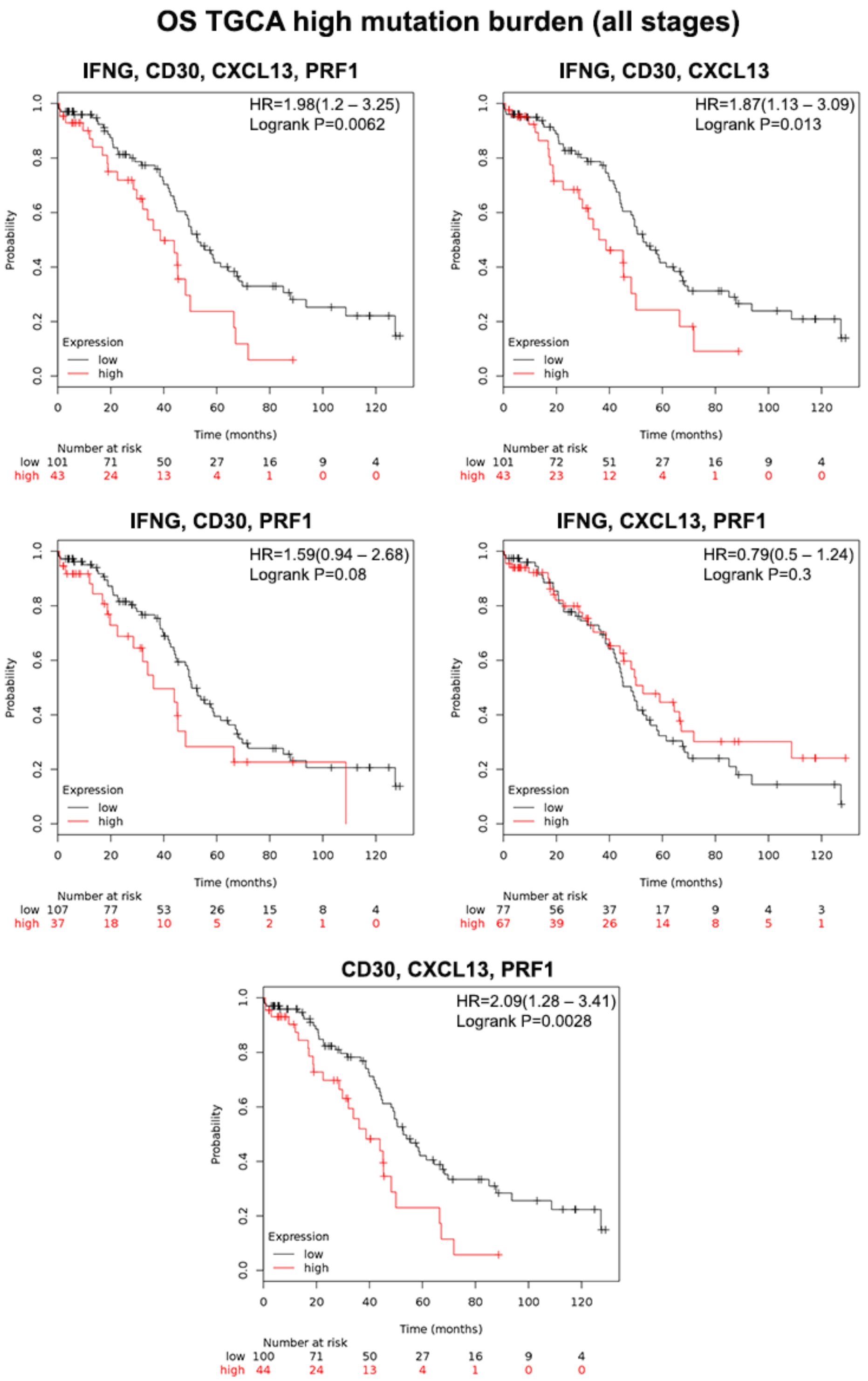

Supplementary Figure 1. Kaplan-Meier survival plots showing the association between the combined gene expression levels (*IFNG, CD30, CXCL13, PRF1; IFNG, CD30, CXCL13; IFNG, CD30, PRF1; IFNG, CXCL13, PRF1;* and *CD30, CXCL13, PRF1*) and prognosis (OS) in with high mutational load ovarian cancer patients from all stages. The hazard ratio (HR) and the Kaplan-Meier *p* value are shown.
